# Synergistic Construction of Sub-Nanometer Channel Membranes through MOF–Polymer Composites: Strategies and Nanofiltration Applications

**DOI:** 10.3390/polym16121653

**Published:** 2024-06-11

**Authors:** Qian Chen, Ying Tang, Yang-Min Ding, Hong-Ya Jiang, Zi-Bo Zhang, Wei-Xing Li, Mei-Ling Liu, Shi-Peng Sun

**Affiliations:** 1 State Key Laboratory of Materials-Oriented Chemical Engineering, National Engineering Research Center for Special Separation Membranes, Jiangsu National Synergetic Innovation Center for Advanced Materials, College of Chemical Engineering, Nanjing Tech University, Nanjing 211816, China; 2 Nanjing Membrane Materials Industrial Technology Research Institute Co., Ltd., Nanjing 211816, China; 3 NJTECH University Suzhou Future Membrane Technology Innovation Center, Suzhou 215100, China

**Keywords:** nanofiltration membranes, polymer, MOF, functional, molecular separation

## Abstract

The selective separation of small molecules at the sub-nanometer scale has broad application prospects in the field, such as energy, catalysis, and separation. Conventional polymeric membrane materials (e.g., nanofiltration membranes) for sub-nanometer scale separations face challenges, such as inhomogeneous channel sizes and unstable pore structures. Combining polymers with metal–organic frameworks (MOFs), which possess uniform and intrinsic pore structures, may overcome this limitation. This combination has resulted in three distinct types of membranes: MOF polycrystalline membranes, mixed-matrix membranes (MMMs), and thin-film nanocomposite (TFN) membranes. However, their effectiveness is hindered by the limited regulation of the surface properties and growth of MOFs and their poor interfacial compatibility. The main issues in preparing MOF polycrystalline membranes are the uncontrollable growth of MOFs and the poor adhesion between MOFs and the substrate. Here, polymers could serve as a simple and precise tool for regulating the growth and surface functionalities of MOFs while enhancing their adhesion to the substrate. For MOF mixed-matrix membranes, the primary challenge is the poor interfacial compatibility between polymers and MOFs. Strategies for the mutual modification of MOFs and polymers to enhance their interfacial compatibility are introduced. For TFN membranes, the challenges include the difficulty in controlling the growth of the polymer selective layer and the performance limitations caused by the “trade-off” effect. MOFs can modulate the formation process of the polymer selective layer and establish transport channels within the polymer matrix to overcome the “trade-off” effect limitations. This review focuses on the mechanisms of synergistic construction of polymer–MOF membranes and their structure–nanofiltration performance relationships, which have not been sufficiently addressed in the past.

## 1. Introduction

The continuous enhancement of separation precision is crucial for the sustainable development of the chemical industry. As separation precision reaches the sub-nanometer scale, due to the confined mass transfer effects, the transport properties of materials exhibit significant differences from those at the macroscopic scale. This distinction provides wide application prospects in fields such as energy, catalysis, and high-value separation. Nanofiltration enables the precise separation of small molecules, ions, and other substances at the sub-nanometer scale, showing vast application prospects in biomedicine, petrochemicals, water treatment, and organic solvent treatment [1,2]. Sub-nanometer pore materials are commonly applied in the form of membranes. In polymeric nanofiltration membranes, sub-nanometer pores are formed by interconnected microvoids, which result from the packing of linear polymers or the network structures of cross-linked polymers [3]. However, due to the randomness in the distribution of polymers, traditional polymeric membrane materials encounter significant challenges, including non-uniform channel sizes and unstable pore configurations, which hinder their separation performance at the sub-nanometer scale [4,5]. Compared to other nanomaterials, metal–organic frameworks (MOFs) possess uniform sub-nanometer channels, which size and surface properties can be precisely regulated. They have garnered widespread attention in the selective separation of small molecules. However, MOFs are predominantly available in powdered form, which undermines the scale application of their sub-nanometer channels.

Inspired by the “rigid bone–flexible muscle” composite system in the human body, combining flexible polymers with rigid MOFs may solve the problem of constructing tunable sub-nanometer channels while also preserving their distinct excellent properties. However, simply mixing polymers with MOFs could not effectively ensure the formation of precise sub-nanochannels. It is vital to thoroughly investigate the unique properties of MOFs and polymers, as well as their structure–performance relationship and interfacial compatibility [6,7]. (1) When MOF polycrystalline membranes are used for sub-nanometer separations, the exposed MOF layer plays a dominant role in the separation performance. However, the growth and surface properties of MOFs are challenging to control precisely. The abundant reactive groups in polymers may selectively capture metal clusters or organic ligands in MOFs, allowing the polymers to accurately dictate the growth and surface properties of MOF polycrystalline membranes. (2) When mixed-matrix membranes (MMMs) are utilized for sub-nanoscale separations, MOFs are integrated into the polymer matrix membranes to serve as additional transport channels. The interfacial compatibility between MOFs and polymers, along with the distribution of MOFs, is crucial in the effective construction of sub-nanochannels. (3) When thin-film nanocomposite (TFN) membranes are applied to sub-nanometer-scale separations, the polymer selective layers formed by interfacial polymerization primarily influence separation, although their performance is often constrained. This limitation arises from the ineffective regulation of the formation process of the selective layer, which, in turn, hampers the construction of uniform sub-nanometer channels. MOFs play dual roles in the process. On the one hand, they act as interlayers to regulate monomer diffusion and facilitate the formation of selective layers. On the other hand, they are dispersed as fillers in the aqueous or organic phase to create uniform sub-nanochannels within the polymer matrix.

**Figure 1:**
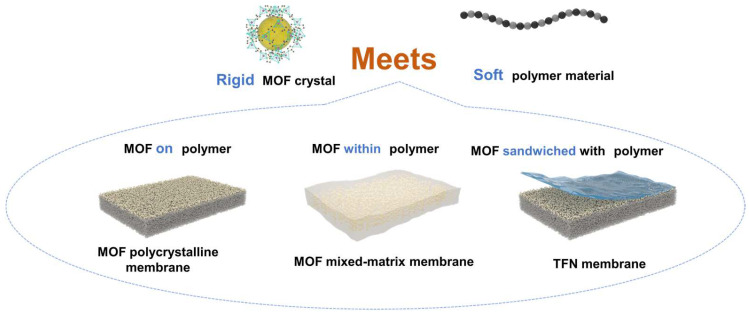
Schematic diagram of sub-nanometer channels formed by MOFs and polymers.

Given the significant impact of different configurations of polymer–MOF membranes on membrane performance, this review systematically introduces their properties, structure, and performance effects based on the spatial relationship between the polymers and MOFs. It categorizes the channels into (1) MOF polycrystalline membranes with MOFs on the polymer surface, (2) MMMs incorporating MOFs into the polymer matrix, and (3) TFN membranes with MOFs sandwiched between the polymer substrate and the selective layer, as well as within the selective layer. Then, the applications of the three different types of sub-nanochannel membranes in water treatment and organic solvent nanofiltration systems are systematically compared. Finally, the challenges and opportunities for the future development of polymer–MOF membranes are highlighted (Figure 1).

## 2. Polymer Regulation of MOF Functionality and Growth

The growth and surface characteristics of MOFs critically influence their separation performance. Therefore, precise control over the growth process and surface properties of MOFs is essential. Although the functionalities of MOFs could be tailored through post-synthetic and organic ligand modifications, these methods are typically intricate and laborious [8]. Hence, there is a need for a convenient and universal strategy to precisely control the growth and surface properties of MOFs. Polymers, with their chains bearing active groups, ionic groups, and conductive groups, possess a wealth of functionalities [9]. Therefore, utilizing polymers to enrich the functionalities of MOFs represents a convenient and universal strategy [10,11]. This discussion will be divided into two points: the effect of different types of polymers (hydrophobic, cationic, anionic, etc.) on the surface characteristics of MOFs and the impact of various polymers on the growth process and morphology of MOF crystals. This discussion aims to clarify how polymers influence the growth process of MOFs and the effects of various polymers on the surface modification of MOFs.

### 2.1. Regulation of MOF Functionality by Polymers

**Figure 2:**
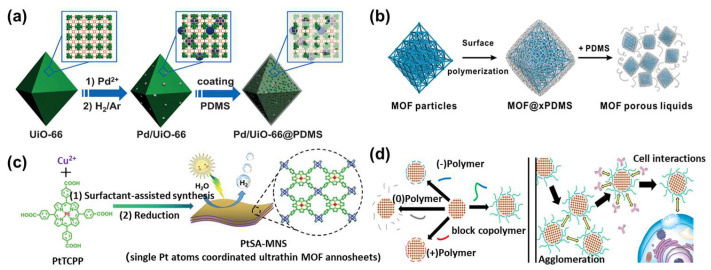
Functionality regulation of MOFs by polymers. (**a**) Hydrophobic modification of UiO-66 via PDMS coating [12], (**b**) transformation of PDMS-modified MOF into a porous liquid [15], (**c**) PVP-regulated photocatalytic performance of MOF nanosheets [17], and (**d**) the surface modification of Zr-fum with polymers possessing varied electrical properties [18].

The surface properties of MOF crystals significantly impact their performance in separating molecules or ions, as well as their long-term stability. If the MOF surface exhibits hydrophobic characteristics, it may reduce the interaction between water molecules and the MOF ligands, thereby enhancing its stability. Jiang et al. deposited the hydrophobic polydimethylsiloxane (PDMS) on the surface of UiO-66 nanoparticles to enhance their hydrophobicity. PDMS is characterized by its low surface energy and excellent hydrophobic properties. Its coating transformed UiO-66 nanoparticles from hydrophilic to hydrophobic, markedly influencing their catalytic efficiency for substrates with different levels of hydrophilicity and hydrophobicity (Figure 2a) [12]. By further adjusting the hydrophilicity and hydrophobicity of sulfonated UiO-66 with PDMS coating, the team enhanced its utility in electrocatalytic reactions. The modified hydrophobic surface promotes the formation of active intermediates and inhibits hydrogen evolution reactions [13]. In addition, PDMS could also act as a solvent, transforming UiO-66 from a solid powder into a porous liquid. Porous liquids are a new type of material that combine the fluidity of liquid adsorbents with the porosity of solid adsorbents, attracting growing attention. However, it is very difficult to maintain porosity while achieving fluidity [14]. Li et al. achieved a porous liquid by uniformly coating UiO-66 surfaces with PDMS through a free radical polymerization technique [15]. The inherent fluidity of PDMS ensures the preservation of the MOF pore structure, thereby forming a porous liquid. This polymer-modified MOF material demonstrates broad application prospects in the field of gas adsorption (Figure 2b). Polymers do not always enhance the performance of MOFs, and sometimes, they may even inhibit and hinder the performance of MOFs. Jiang et al. utilized another polymer, polyvinylpyrrolidone (PVP), to encapsulate UiO-66-NH_2_ loaded with Pt nanoparticles for photocatalytic hydrogen production tests [16]. However, introducing PVP hindered the electron transfer between Pt and MOF, reducing the photocatalytic activity of the catalyst. Zuo et al. employed PVP to regulate the thickness of porphyrin-based MOF nanosheets. PVP selectively adheres between layers of nanosheets, thus facilitating their dispersion and promoting anisotropic growth in two dimensions. This interaction results in the formation of ultrathin nanosheets with a thickness of approximately 2.4 nm. By leveraging the excellent processability of two-dimensional layered materials, the PVP-modified porphyrin-based MOF nanosheets could further be dispersed in ethanol and then drop-cast into films, demonstrating a superior photocatalytic hydrogen production performance (Figure 2c) [17]. MOFs are not only pivotal in catalysis but also hold significant applications in biomedical diagnostics and therapeutics. The surface chemistry of MOFs significantly influences their interaction with biological systems, making controlled regulation essential. Zimpel et al. conducted an initial systematic investigation into how MOF surface functionality interacts with biological interfaces, such as cell binding and protein adhesion [18]. They systematically utilized cationic polymers (branched polyethyleneimine (PEI) and dendritic fourth-generation PAMAM), anionic polymers (polyglutamic acid (PGlu) and polyacrylic acid (PAA)), non-ionic polymers (PEG and polysorbitol ester), and block copolymers (polyglutamic acid salt-b-polysarcosine (pGlu-PSar)) for the surface modification of Zr-fumarate. The results demonstrate that different polymers could attach to the MOF surface by exchanging their coordinating groups with the modulator (formic acid) in Zr-fum. Polymers containing carboxylic acids and amines significantly enhance the stability of MOFs (Figure 2d).

### 2.2. Regulation of MOF Growth by Polymers

Polymers could also directly participate in the growth and formation processes of MOFs [19]. Currently, the synthesis of MOFs predominantly utilizes conditions that require organic solvents. How to reduce or even eliminate the use of organic solvents in the synthesis of MOFs represents an emerging research direction. Deep eutectic solvents have garnered significant attention due to their green raw materials and their ease of functionalizing MOFs [20]. Due to the inherent ability of polymers to interact with both metal ions and ligands, they hold a significant role in the green synthesis of MOFs [21,22].

**Figure 3:**
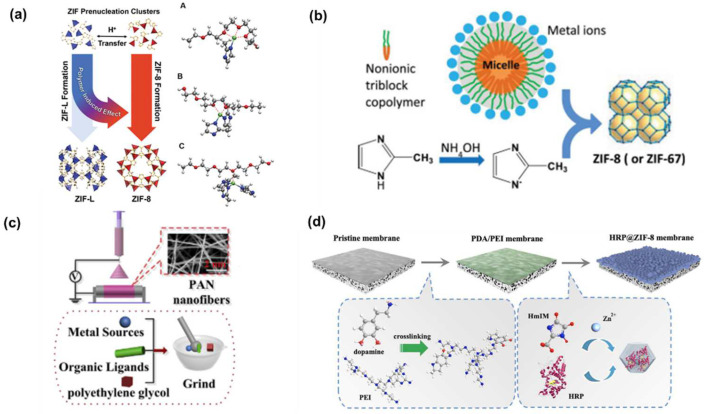
Growth and structure regulation of MOF by polymers. (**a**) Influence of PEO on the growth pathway of ZIF [25], (**b**) effect of the block copolymer on the growth pathway of ZIF-8 [26], (**c**) schematic of PEG-assisted ZIF growth on a nanofiber substrate [27], and (**d**) PDA/PEI-induced cogrowth of MOF and enzymes [28].

Polyethylene glycol (PEG) is a non-ionic surfactant that also serves as a reaction medium for MOF crystals. PEG possesses the following characteristics: (1) it is non-toxic and degradable, approved by the United States Food and Drug Administration for use as an additive in food and cosmetics; (2) the polymer chain of PEG contains many oxygen atoms, which could bind more effectively with metal ions and control the growth of MOFs. Xiong et al. utilized transition metal ions (Fe^2+^, Co^2+^, Zn^2+^, and Cd^2+^) and ligands such as 1,4-benzenedicarboxylic acid (BDC) or isophthalic acid (IPA) in coordination with PEG to synthesize eight new types of MOFs [23]. It is important to note that the crystals could not be prepared without using PEG as a solvent. Subsequently, they also reacted four different types of transition metal acetates (Co, Mn, Ni, and Zn) with trimesic acid (BTC) and pyridine (py) in the presence of PEG, resulting in the preparation of four new types of 3D MOFs [24]. However, the above studies did not explore the role of the polymer in MOF growth. Gaurav et al. carried out experimental and theoretical studies on the crystallization of zeolitic imidazolate frameworks (ZIFs) with PEG present. In the absence of PEG, ZIF formation only occurred when the ligand-to-metal salt ratio exceeded 20. Surprisingly, with PEG present, ZIF was able to form at a ligand-to-metal salt ratio greater than 8 [25]. This could be attributed to PEG chains binding with Zn nodes within the pre-nucleation clusters of ZIF, thereby altering the growth process and enabling ZIF formation at lower ratios of ligand to metal salt (Figure 3a). Yao et al. separately dissolved the ZIF precursor, a Zn metal salt, and the ligand 2-methylimidazole (2-Hmim) in water. They then introduced a triblock copolymer surfactant into the aqueous solution of the Zn metal salt. PEO groups on the surface of the triblock copolymer could adsorb metal ions, thus facilitating the formation of ZIF. However, if the polymer is absent from the precursor solution, the metal ions in water would directly interact with deprotonated imidazole to form via Zn structures, preventing the formation of the ZIF structures (Figure 3b) [26]. By leveraging the strategy of PEG-assisted growth of ZIFs, Peng et al. coated nanofiber surfaces with a PEG solution containing ZIF precursors [27]. They then employed a hot-pressing method to facilitate the growth of ZIF, applying it for the efficient removal of heavy metal Cu^2+^ from aqueous solutions (Figure 3c). Not limited to PEG, other polymers also possess characteristics that control the crystallization process of MOFs. Xu et al. induced the cogrowth of MOFs and enzymes by coating membrane surfaces with polydopamine (PDA) and polyethyleneimine (PEI), constructing biomimetic catalytic membranes for the removal of bisphenol A from water [28]. PDA reacted with PEI through a Michael addition or Schiff base reactions, serving as a connector for the growth of MOFs and membranes. This interaction resulted in the in situ formation of a continuous enzyme@ZIF layer structure on the membrane surface (Figure 3d). Polymers not only regulate the growth process of MOFs but also influence their surface morphology. Uemura et al. used poly(vinylsulfonic acid, sodium salt) (PVSA) to regulate the size and shape of Cu-based MOFs. The size of the MOF particles increases proportionally with the concentration of PVSA in the precursor, reaching sizes of up to 70 μm. The increased crystal size is a result of electrostatic interactions between PVSA and Cu precursors, which reduces the number of MOF nucleation sites [29].

Polymers could interact not only with metal ions but also with organic ligands to regulate the crystallization process. Baiker et al. employed poly(acrylic acid, sodium salt) (PAA) to control the particle size of Cu-BTC, reducing the size of the MOF from micrometers to nanometers. The size reduction is attributed to PAA, which accelerates the deprotonation of the BTC ligand, thereby increasing the nucleation rate and resulting in smaller crystal morphologies. Although the MOF crystals became smaller, the expanded surface area enabled Cu-BTC to adsorb twenty times more dibenzylamine compared to the original MOF [30]. Beyond influencing the crystallization process of MOFs, polymers could also affect the pore structure of MOFs during their growth. Li et al. introduced PVP into the precursors of Zr-based MOFs to construct hierarchical pore structures consisting of macropores, mesopores, and micropores [31]. Like the previously mentioned examples, MOF crystals cannot form in the absence of PVP. Remarkably, the addition of PVP led to the formation of irregular MOF structures and macropores after 2 h of heating. Upon extending the heating duration to 6 h or more, continuous macropores and hierarchical pore structures developed. Furthermore, this method showcases its universality through the synthesis of diverse MOF materials, including UiO-66, NU-1200, NU-1000, and PCN-777.

## 3. Strategies for the Preparation of MOF–Polymer Membranes

As previously mentioned, the interaction between MOFs and polymers can result in three types of membranes: MOF polycrystalline membranes, MMMs, and TFNs. These three different configurations of membranes face both common issues (interfacial compatibility) and unique challenges. Therefore, it is necessary to elucidate and compare these issues and their solutions to clarify their structure–performance relationships. For MOF polycrystalline membranes, the MOF layer has a critical impact on their separation performance. The primary challenges include the difficulty in controlling the crystal plane, defects, and thickness of MOFs. Additionally, poor interfacial compatibility manifests as weak adhesion between MOFs and the substrate in polycrystalline membranes. Here, we discuss the regulation of the crystal plane, surface properties, morphology, and defects of MOF membranes by polymers and how these adjustments affect the separation performance of the membranes. For MOF mixed-matrix membranes, the primary issue is poor interfacial compatibility [32]. To address these challenges, this section introduces methods and strategies for modifying both polymers and MOFs. For TFNs, the main challenges stem from the difficulty in controlling the growth process of the polymer selective layer and overcoming the “trade-off” effect. This section introduces strategies for using MOFs as an interlayer to regulate the polymer selective layer and methods to incorporate MOFs as transport channels to overcome the “trade-off” effect. By analyzing and comparing these methods, we further clarify the structure–performance relationship of MOFs and polymers, guiding the preparation of high-performance nanofiltration membranes applied in sub-nanometer channel transport.

### 3.1. MOF Polycrystalline Membranes

The performance of MOF membranes is often limited by intercrystalline defects. Polymers can reduce these defects through the following approaches: (1) pre-binding with metal ions in MOF precursors to provide anchors for subsequent growth; (2) regulating the MOF crystallization process to form thinner membranes, thereby reducing defects; and (3) embedding metal ions in advance to act as a “sustained release layer” for subsequent MOF growth.

**Figure 4:**
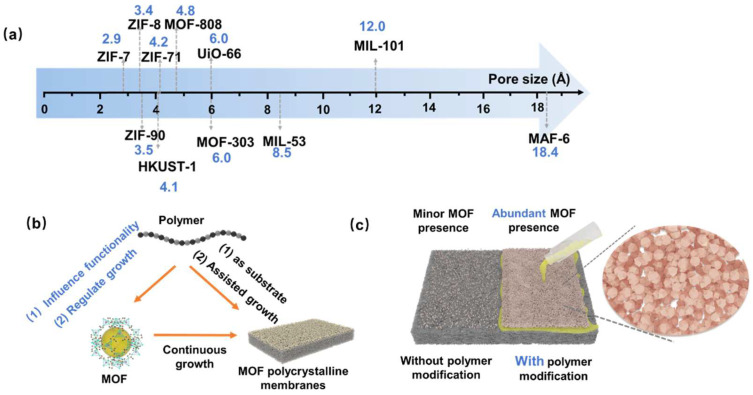
(**a**) Diagram of the pore sizes of common water-stable MOFs; (**b**) schematic of the interrelationship among MOFs, polymers, and the MOF polycrystalline membrane; and (**c**) schematic comparison of MOF growth on substrates with and without polymer modification.

Polymers not only modify the functionality of MOFs and regulate their growth processes but also play a crucial role in the transition from MOF crystals to MOF membranes. Due to their inherent uniform channels, ranging from angstrom to nanometer levels, MOFs could facilitate precise molecular or ion sieving (Figure 4a). However, the polycrystalline MOF membranes often exhibit intercrystalline defects or cracks, and there also exists a “trade-off” effect between MOF crystallization and membrane formation. In other words, forming a defect-free membrane structure becomes more challenging as the crystallinity of the crystal increases. MOF polycrystalline membranes refer to the growth of MOF crystals on a porous substrate to form interconnected MOF layers, where the MOF layer provides a sieving function [33]. The porous substrates could be divided into inorganic (alumina, metal mesh, etc.) and polymer materials (polyimide, nylon, etc.). The growth of polycrystalline MOF membranes is consistently challenging, requiring remedies for concerns regarding MOF growth and the interface adhesion between MOFs and the substrate. Polymers, rich in active functional groups, may effectively modify the substrate material to improve the affinity between MOFs and the substrate. Additionally, the introduction of polymers could regulate the crystallization process of MOFs, as mentioned earlier. Therefore, incorporating polymers in the preparation process of MOF polycrystalline membranes is expected to solve these problems. Figure 4b illustrates the interrelationship among MOFs, polymers, and polycrystalline MOF membranes. Here, the preparation strategies of MOF polycrystalline membranes based on different substrate materials (inorganic and polymer types) will be introduced.

#### 3.1.1. Growth of MOFs on the Inorganic Carriers Modified with Polymers

Inorganic substrates, with their robust mechanical properties, high-temperature resistance, and tolerance to organic solvents, provide an optimal environment for the growth of MOF polycrystalline membranes. However, the absence of growth sites between inorganic substrates and MOFs leads to weak binding forces between them. Researchers often employ methods such as secondary growth and surface modification to enhance this interaction [34,35].

**Figure 5:**
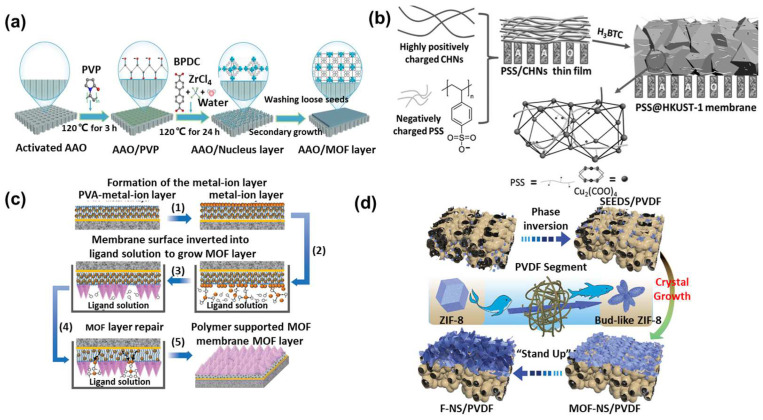
Effect of polymers on the growth of the MOF polycrystalline membrane. (**a**) PVP-modified AAO substrate to promote UiO-67 growth [37], (**b**) PSS aiding HKUST-1 growth on AAO [39], (**c**) PVA mending MOF defects for defect-free membranes [44], and (**d**) the “MOF on MOF” strategy for in situ MOF membrane growth [47].

The abundant functional groups in polymers not only offer sites for MOF growth but also modify the inorganic substrates, serving as adhesives between the inorganic substrates and MOFs (Figure 4c). Liu et al. employed PVP to modify alumina substrates, subsequently achieving the in situ growth of MOF-808 on the substrate for dye/salt separation applications. Compared to the initial alumina substrates, the abundance of oxygen-containing active groups in PVP facilitated a more effective binding with Zr metal clusters, enhancing the nucleation density of MOF-808 on the support. Moreover, the unique large pore structure of MOF-808 allowed for efficient salt permeation while intercepting dyes, showcasing a high salt/dye selectivity of 287.3 and permeance of 4.37 L m^−2^ h^−1^ bar^−1^ (Figure 5a) [36]. Beyond improving the bond between MOF and alumina substrates, PVP also allows for the orientation control of MOFs. Zhang et al. utilized PVP for the in situ growth of UiO-67 on anodic aluminum oxide (AAO) substrates. Similar to the previous instance, the affinity of active groups in PVP for Zr^4+^ ensured the uniform anchoring of Zr^4+^ ions on the AAO substrate. Subsequently, these ions coordinated with 4,4′-biphenyl dicarboxylic acid (BPDC) ligands, leading to the formation of UiO-67 layers with an [022] orientation [37]. The unique orientation of the UiO-67 membranes facilitated the rapid passage of Li^+^, resulting in a high Li^+^/Mg^2+^ separation ratio of 159.4.

The thickness of the MOF polycrystalline membranes significantly influences their separation performance. However, conventional methods struggle to precisely control this thickness. As mentioned before, polymers have dual effects on MOF membrane growth, promoting or restricting it. Currently, the thickness of MOF polycrystalline membranes used in separation applications tends to exceed 1 μm. Although thicker MOF membranes exhibit fewer defects, their considerable thickness limits the effective mass transfer. Using polymers to control MOF crystallization may construct ultrathin MOF membranes. Jiang et al. introduced the polymer PEG-NH_2_, which is capable of chelating with Zn^2+^, into the ZIF precursor and synthesized ZIF-8 via an electrochemical approach. Lone pair electrons on PEG’s amino and ether oxygen groups compete with 2-methylimidazole for Zn^2+^ coordination, reducing ZIF crystallization and leading to thinner ZIF layers [38]. Polymers play a nuanced role in not only regulating the growth of MOF membranes but also modifying MOF surfaces, which affects the separation performance. Peng et al. applied sodium polystyrene sulfonate (PSS) onto AAO substrate embedded with copper hydroxide nanowires, followed by the in situ growth of HKUST-1 on this substrate. The sulfonate groups present in PSS exhibit varying affinities for Li^+^, Na^+^, K^+^, and Mg^2+^, leveraging these differences to achieve the efficient separation of mono/divalent salts. The membrane exhibited ideal selectivities of 78 for Li^+^/Na^+^, 99 for Li^+^/K^+^, and 10,296 for Li^+^/Mg^2+^, respectively. The actual binary ion selectivities were 35 for Li^+^/Na^+^, 67 for Li^+^/K^+^, and 1815 for Li^+^/Mg^2+^, respectively (Figure 5b) [39].

#### 3.1.2. Growth of MOFs on the Polymer Substrates

Polymer supports are cost-effective, highly flexible, and easy to scale up. However, the conditions of MOF growth typically involve organic solvents and high temperatures, where common polymer materials may not endure. Furthermore, the nucleation and growth of MOFs on polymer substrates is also challenging. To enhance the bond between MOFs and polymer supports, Yao et al. employed the contra-diffusion synthesis method to create defect-free ZIF-8 films on nylon membranes. This method placed the metal salt and organic ligand solutions on opposing sides of a nylon polymer substrate, enabling mutual diffusion to form a ZIF film. The chemical potential difference between the solutions drove the diffusion process towards the polymer substrate. As the reaction occurs not just on the surface but also within the pores, the binding force between the MOF and the polymer is enhanced. The contra-diffusion growth method is characterized by its “healing” and “self-limiting” nature. The term “healing” refers to the process where solutions diffuse through intercrystalline gaps, facilitating the growth of ZIF that subsequently fills these spaces. As the gaps become filled and crystals fully form, the diffusion of the precursor solution ceases, thereby halting the reaction. This phenomenon demonstrates the “self-limiting” characteristic of this method [40,41]. Enriching metal ions on the surface of a porous substrate is a universal strategy for constructing high-quality MOF polycrystalline membranes. Phytic acid, known for its strong metal ion chelating capabilities, could be utilized in the preparation of MOF polycrystalline membranes. Jiang et al. first proposed the preparation strategy for “metal–organic phosphoric acid” membranes in 2019, using phytic acid as an electron donor and transition metal ions (Ag^+^, Zn^2+^, Ni^2+^, Fe^3+^, and Zr^4+^) as electron acceptors [42]. According to acid–base coordination theory, only hard acids (Fe^3+^, Zr^4+^) and similarly hard base phosphoric acid ligands could combine to form defect-free membrane structures. Jiang et al. then used pre-embedding metal ions on polymer surfaces to serve as nucleation sites for MOFs, thereby improving the MOF–polymer substrate bond [43]. They subsequently utilized contra-diffusion synthesis for MOF membrane preparation. Metal–organic phosphoric acid could self-assemble into a coating on the polymer substrate, enhancing the enrichment of metal ions and facilitating MOF growth. This strategy works for various polymers (PES, PAN, and HPAN) and MOFs (e.g., ZIF-8 and HKUST-1). This indicates that the influence of metal ions on MOF growth is exceptionally subtle. Researchers have also utilized the characteristic of polymers to adsorb metal precursor ions, which helps to compensate for defects between MOF crystals, enabling the fabrication of large-scale, defect-free membranes. Yu et al. initially coated a polysulfone (PSF) substrate with a PDMS layer to create a uniform surface for MOF growth [44]. They then immersed the PDMS-layered membrane in a PVA–metal ion solution to serve as a “release layer” for subsequent MOF growth. Subsequently, the membrane was immersed in metal ion and organic ligand solutions, leading to MOF formation upon the interaction of solution ligands with membrane surface metal ions. However, metal ions in the pure metal ion layer partially dissolved into the solution, causing defects in MOF growth. Surprisingly, metal ions in the release layer were gradually and consistently supplied to the defect sites, facilitating MOF growth and self-repair in these areas. During this process, ligand molecules were attracted to the PVA–metal ion layer. As mentioned before, polymers significantly affect the MOF crystallization process. In this case, the PVA–metal ion layer greatly promotes the deprotonation step of MOF, facilitating rapid nucleation. Furthermore, this method enabled the preparation of membranes with a packing area of 4800 cm^2^, demonstrating the practical application prospects of polymer-assisted MOF growth strategies in developing large-area MOF membranes (Figure 5c). Additionally, Yu et al. added Ag^+^ to the pure metal ion solution, leveraging the oxidizing and etching effects of Ag^+^ solution to create hierarchical porous membranes with micro- and mesopores [45].

Unlike MOF particles, two-dimensional MOF nanosheets possess ultrathin thickness and an exceptionally large surface area, making them advantageous for membrane separation applications due to relatively fewer lattice defects [2]. To tackle disorderly stacking and microporous defects in Cu-TCPP two-dimensional nanosheets, Chen et al. employed the “MOF on MOF” strategy, enhancing the triple-nozzle electrostatic spraying technique [46]. One nozzle dispersed Cu-TCPP nanosheets, while the other two separately sprayed Cu-TCPP metal cluster and organic ligand solutions. This approach not only facilitates a more precise application but also contributes to a tighter integration with the preceding triple-nozzle technique. Covalent bonds between the secondary MOF particles and nanosheets filled the gaps, reducing defects and resulting in more orderly nanosheet stacking. The membrane achieved a retention of over 90% for rose red dye, with an ethanol permeance of around 90 L m^−2^ h^−1^ bar^−1^. However, electrostatic spraying also poses challenges for scaling up, particularly in producing membranes with larger pores that struggle to retain small molecules and ions. Zhao et al. improved MOF nanosheet adhesion to PVDF substrates by integrating ZIF-8 crystals into the PVDF casting solution [47]. Introducing ZIF-8 created heterogeneous nucleation sites and strong binding forces for the subsequent growth of MOF nanosheets. By flipping, twisting, and shaking the membrane substrate, the nanosheets were securely attached without tearing (Figure 5d). In contrast to the secondary growth strategy, Zhao et al. synthesized MOF nanosheets with excellent self-film-forming properties, offering a new approach for preparing large-area and ultrathin MOF membranes. In 2017, Zhao et al. initially synthesized NUS-8 nanosheets via a modulated hydrothermal method. NUS-8 nanosheets are composed of Zr_6_O_4_(OH)_4_ or Hf_6_O_4_(OH)_4_ clusters and 1,3,5-benzenetriphosphonate (BTB^3−^) ligands. Unique heterogeneous synthesis conditions restrict planar ligands to interface reactions and crystal growth, allowing for the creation of two-dimensional NUS-8 materials [48]. In 2021, Zhao et al. optimized the synthesis by using formic acid as capping molecules. Formic acid molecules selectively coordinated with Zr^4+^ sites, forming multi-layered NUS-8 nanosheets with superb solution processability. This allowed the preparation of high-performance aerogels, xerogels, and large-area thin films [49]. In 2023, Zhao et al. used a scraping and evaporating method to produce NUS-8 membranes with variable thickness and uniformity on PAN-400 substrates. The excellent adhesion, flexibility, and processability of NUS-8 nanosheets ensured a strong binding to PAN substrates. The membrane achieved nearly 98% retention of common Mg^2+^, Al^3+^, and dyes, with a permeance of 1 to 3 L m^−2^ h^−1^ bar^−1^ [50].

### 3.2. Growth of MOFs within Polymers: MOF MMMs

**Figure 6:**
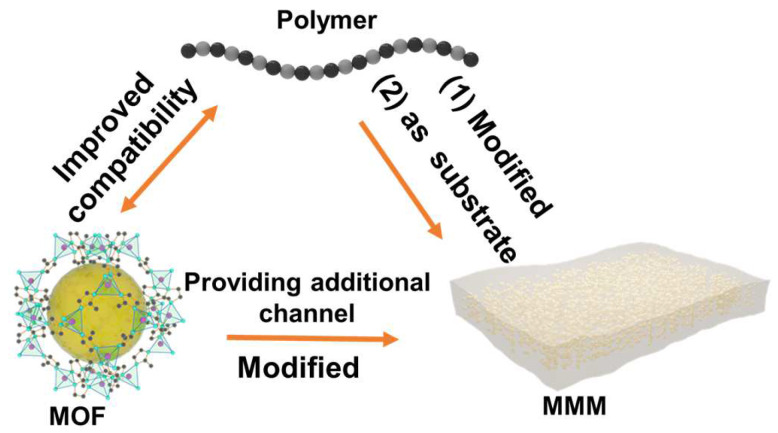
Schematic of the interrelationship among MOFs, polymers, and MMMs.

MOF polycrystalline membranes are formed by growing MOFs on the porous carrier, whereas MOF MMMs are prepared by embedding MOFs within the polymer. MMMs are typically fabricated through a blending technique. This method involves dissolving the polymer in a solvent to form a casting solution. Then, MOF particles are added into the casting solution and stirred or sonicated to achieve uniform dispersion. Subsequently, methods such as phase transformation are employed to remove the solvent from the casting solution and form a membrane. The organic–inorganic hybrid nature of MOFs enhances polymer compatibility compared to conventional inorganic materials. However, challenges such as MOF particle aggregation within the polymer matrix and less-than-ideal MOF–polymer interfacial compatibility still exist. Benefiting from the tunable properties of MOF materials, modifications could be carried out to improve the interface between MOF materials and polymers. The relationship among MMMs, MOFs, and membranes is illustrated in the following Figure 6.

**Figure 7:**
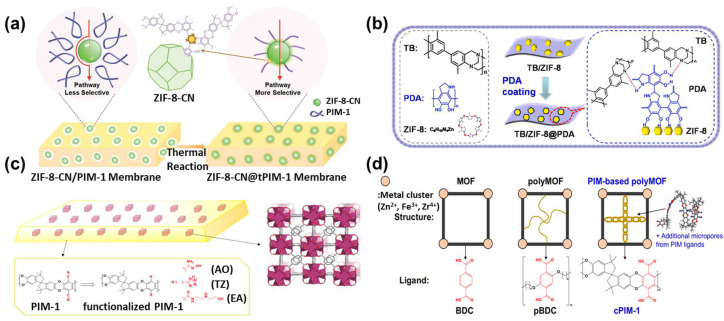
Strategies for improving the interface compatibility of MOF mixed-matrix membranes. (**a**) -CN-modified ZIF for mixed-matrix membrane fabrication [51], (**b**) PDA-modified ZIF-8 for well-compatible mixed-matrix membrane fabrication [53], (**c**) functionalized polymers for mixed-matrix membrane construction [57], and (**d**) schematic of the polyMOF growth strategy [58].

To address the issue of interfacial compatibility between MOFs and polymers in mixed-matrix membranes, several approaches are commonly employed: modification of MOFs (including functionalization of MOFs and the “MOF on MOF” strategy), modification of polymers, and in situ growth methods. A common method for modifying MOFs is the modification of their ligands. Zhu et al. improved the compatibility between ZIF-8 and PIM polymers using a mixed-ligand approach in ZIF-8 synthesis. They incorporated -CN groups into the ZIF-8 structure using 4,5-dicyanoimidazole as an auxiliary ligand. After thermal activation, ZIF-8-CN was further covalently bonded with PIM-1, significantly improving the interface compatibility between ZIF-8 and the PIM polymer (Figure 7a) [51]. As mentioned before, polymers could also functionalize MOFs, which is simpler compared to ligand modification methods. Cseri et al. adopted a polymer modification approach for MOF modification, coating aminated UiO series with poly (N-isopropyl acrylamide) (PNIPAM) [52]. This modification increases the MOF–polymer matrix compatibility, significantly enhancing the organic solvent nanofiltration performance. Specifically, it increased acetone solvent permeability, with a molecular weight cutoff of 160–290 g/mol. Fan et al. used PDA to modify MOFs, boosting the interfacial compatibility with Tröger base polymer membranes. The NH and hydroxyl groups in PDA molecules form multiple hydrogen bonds with the Tröger base polymer. Additionally, the flexibility of PDA partly reduces interface stress, thus enhancing the mechanical properties of the membrane (Figure 7b) [53].

A larger interface contact area between MOFs and polymers generally suggests improved interfacial compatibility. However, the relatively large size of MOF particles often results in limited contact with polymers. Wu et al. utilized a “dual-interface approach” for enhanced compatibility. This method involved the growth of smaller MOF-74 particles, approximately 20 nm in size, on the surface of original MOF particles around 170 nm. The reduced size and open active sites of MOF-74 particles expanded the interface area and tripled the contact interaction force with the polymer [54]. Zhang et al. adopted a similar strategy by growing smaller Ag_4_tz_4_ particles (10 nm) on larger ZIF-67 (120 nm) to form a ZIF-67@Ag_4_tz_4_ core–shell structure [55]. The inclusion of Ag_4_tz_4_ not only strengthens the interfacial bonding with the polymer but also significantly mitigates the agglomeration of ZIF-67. By leveraging hydrogen bonding, Li et al. skillfully adjusted the compatibility between ZIF and glassy polymer 6FDA durene and rubbery polymer Pebax 1657 [56]. This approach smartly used the basicity of dimethylimidazole in ZIF ligands to manage the hydrolysis/condensation of phenolic resins. The phenolic hydroxyl groups of resin formed hydrogen bonds with the C=O groups of the polymer, resulting in a core–shell structure with ZIF that greatly improved their interfacial compatibility.

Functionalization of MOFs could indeed enhance their interfacial compatibility with polymers. However, modifying MOFs may alter or block their porous structure, thereby affecting their separation performance. Alternatively, modifying polymers represents another approach to improve interfacial compatibility. Carja et al. functionalized PIM-1 with aminooxy, tetrazole, and N-((2-ethylamino)ethyl)amide groups to compare their interactions with UiO-66 [57]. Among these, the aminooxy-functionalized PIM-1 demonstrates the strongest interfacial interaction with the MOF surface. This strong interaction is likely due to the hydrogen bonding between the O(OH) groups of AO-PIM-1 and the H(OH) on the surface of UiO-66 (Zr), which simultaneously promotes the adhesion between MOF nanoparticles and the polymer matrix (Figure 7c). Functionalization of MOFs and polymers markedly enhances their interfacial compatibility, which is vital for boosting the mixed-matrix membrane performance. Beyond simple modifications to polymers, introducing MOF ligand units into the polymer structure could also enhance interfacial compatibility. Lee et al. combined carboxylated PIM-1 with UiO-66 to prepare mixed-matrix membranes [58]. Carboxylated PIMs, which contain phthalic acid units, align with the structural characteristics of UiO-66’s organic ligands. The BDC units within the PIM are able to coordinate with metal ions, facilitating the formation of a polyMOF structure. Concurrently, the phthalic acid units can react with metal clusters to generate UiO-66 nanoparticles. This synergistic interaction enhances the interfacial compatibility and dispersion between the PIM and the MOF, resulting in a uniformly integrated composite material (Figure 7d).

The above methods enhance the interfacial compatibility between MOFs and polymers through functionalization. Typically, this process involves dispersing synthesized MOFs into a polymer solution, which is often cumbersome and time-intensive. Altering the procedure to enable the in situ growth of MOFs within the polymer matrix could significantly reduce the preparation time for MOF membranes and improve compatibility. Unfortunately, the in situ synthesis of MOFs within polymers generally requires the alignment of the polymer dissolution system with the MOF growth system. Seoane et al. employed tetrahydrofuran to dissolve MIL-68 precursors and PSF, utilizing a one-pot method to prepare MOF mixed-matrix membranes [59]. This technique eliminates the requirement to dry MOF crystals before dispersion, thereby improving their dispersion and interfacial compatibility. Li et al. adopted a “constrained swelling coupled with solvent-induced crystallization” strategy for the rapid in situ synthesis of ZIF-8 within a polymer substrate [60]. This strategy entailed dissolving ZIF precursors in methanol and mixing them with a PEO polymer to create a casting solution. Subsequently, UV light was used to initiate the formation of the PEO film, which was then immersed in a mixture of ammonia and methanol. Ammonia can accelerate the deprotonation of 2-hmim and facilitate rapid ZIF crystallization, while methanol serves as the dispersant and solvent for ZIF. This method allowed for a maximum ZIF loading of 63.5%. Chen et al. selected PEG, renowned for its pH stability, as the matrix for MOF in situ growth [61]. The flexibility of PEG chains alleviates the precipitation of MOF particles. They employed spin-coating to deposit the polymer and MOF precursor onto a substrate, followed by reacting it with vaporized ligands to form the MOF. This technique resulted in membranes with a thickness of merely 50 nm. Specifically, the growth of ZIF materials in PIMs also faces challenges due to incompatible solvent requirements: water or alcohol is needed for ZIF synthesis, while chloroform is required for PIM dissolution. Inspired by the symbiotic relationship between rhizobia and legume roots, Shao et al. developed a “chloroform–water” symbiotic solvent system for PIM and ZIF materials [62]. Initially, the PIM was dissolved in chloroform, followed by the addition of metal salt and ligand aqueous solutions, creating a uniform oil–water mixture. The compatibility between chloroform and water facilitated an even dispersion of ZIF within the PIM. Furthermore, the presence of chloroform delayed ZIF crystallization, allowing for a more uniform dispersion. This technique drastically cut down MOF post-synthesis processing and greatly enhanced MOF nanoparticle dispersion, achieving a loading capacity of up to 70%.

### 3.3. MOF Sandwiched by Polymers: TFN Membranes

**Figure 8:**
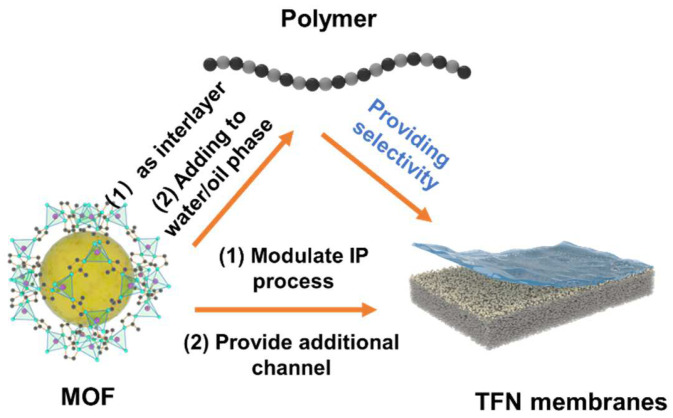
Schematic of the interrelationship among MOFs, polymers, and TFN membranes.

The previously mentioned MOF polycrystalline membranes and MOF mixed-matrix membranes both utilize polymers as carriers, while MOFs serve as sub-nanometer channels to enhance the separation performance. Polymers themselves could also form sub-nanometer channels to provide selectivity, such as through interfacial polymerization. In 1959, Wittbecker et al. synthesized high molecular weight polymers using acyl chloride and compounds with active hydrogen atoms (-OH, -NH, and -SH). Since this reaction occurs at the liquid–liquid interface, it is known as interfacial polymerization. Interfacial polymerization features mild reaction conditions and short reaction times [63]. In 1978, Cadotte’s team used piperazine (PIP) and trimesoyl chloride (TMC) monomers to create the first thin-film composite (TFC) nanofiltration membrane via interfacial polymerization on a PSF substrate. The interfacial polymerization method demonstrates significant advantages in preparing sub-nanometer channel membranes, such as low cost, ease of operation, and suitability for continuous production. The polymeric substrate, drying time, and coating method all have significant impacts on the rejection performance [64]. However, traditional interfacial polymerization technology struggles to overcome the limitations of the “trade-off” effect. MOF crystals have regular pore structure and tunable properties, potentially overcoming the “trade-off” effect. However, MOF crystals often feature structural defects and lack flexibility. Integrating MOFs with interfacial polymerization could address their mutual shortcomings, facilitating the creation of TFN membranes [65]. The preparation of TFN membranes combining MOFs with interfacial polymerization could be divided into three types: as an interlayer, doped into the water/organic phase, and participating in the interfacial polymerization process (Figure 8).

#### 3.3.1. MOF as an Interlayer for Interfacial Polymerization

**Figure 9:**
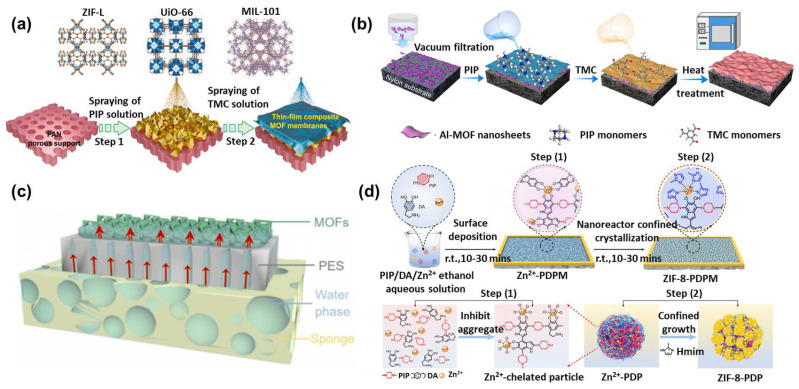
Preparation of TFN membranes involving MOFs. (**a**) Thin-layer MOF composite membranes fabricated by spray deposition [67], (**b**) Al-MOF nanosheets as an interlayer for TFN membrane fabrication [70], (**c**) schematic of TFN membrane preparation assisted by capillary forces [72], and (**d**) TFN membrane fabrication through polymer nano-ion interfacial confinement crystallization [83].

The synergistic design of interfacial polymerization layers is considered one of the most important areas of research in nanofiltration membranes. In 2015, Livingston introduced the concept of TFN membranes with an interlayer, yielding ultrathin, high-flux membranes. Nanoparticles serve as an interlayer to control amine monomer adsorption and release, regulate interfacial polymerization, and enhance the effectiveness of interfacial degassing via nanobubbles [66]. The incorporation of organic groups in MOFs enhances their compatibility and controllability compared to inorganic materials. Zhao et al. dispersed amino group MOF particles (e.g., UiO-66-NH_2_ and MIL-101-NH_2_) in ethanol, combined the dispersion with a PEI aqueous solution, and sprayed it onto a PAN substrate to create a MOF layer. Then, they sprayed a TMC solution on the MOF layer. TMC can react with PEI and the amino groups of MOFs, embedding MOF particles within the PEI–TMC polyamide network. This integration serves to prevent particle leaching and aggregation (Figure 9a) [67]. This membrane exhibited a rejection rate of over 99% for methylene blue and a pure water permeance of 20 L m^−2^ h^−1^ bar^−1^. It greatly exceeds the permeance of commercial nanofiltration membranes. The improvement in performance can be attributed to the amino functional groups in MOFs, which relax the polyamide network and expand the water transport channels due to the intrinsic porosity of MOFs. Besides serving as an interlayer to adjust interfacial polymerization properties, MOFs could also influence the polymerization formation process via their unique characteristics. Peng et al. utilized Fe-TCPP, a MOF known for its photothermal effect that converts light to thermal energy, as an interlayer. The heat quickened the diffusion of monomers and thermally cured the polymerization layer, resulting in a cross-linked, hydrophilic polyamide selective layer. This membrane achieved a permeance of 120 L m^−2^ h^−1^ bar^−1^ for methanol and 160 L m^−2^ h^−1^ bar^−1^ for acetonitrile, while maintaining a 97% rejection rate for acid red [68].

The role of MOF particles as an interlayer in controlling the interfacial polymerization process has been widely reported. However, due to the dispersion and diversity of nanoparticles, the relationship between the properties of the interlayer and performance of the membrane has been challenging to elucidate. The unique configuration and properties of two-dimensional nanosheets provide distinct advantages in TFN membrane preparation. By leveraging the adjustable thickness of interlayers from 2D materials, Xia et al. used the series resistance model to elucidate how the structure of layers affects the membrane resistance distribution and permeability [69]. Initially, Cu-TCPP nanosheets with varying thicknesses were prepared on a PES substrate through vacuum filtration. After using nanosheets as an interlayer for interfacial polymerization and analyzing water resistance with the series resistance model, the study found that an increased thickness of the Cu-TCPP layer decreased the resistance of the polyamide layer. Moreover, increasing the nanosheet thickness initially raised and then lowered the cross-linking degree of the polyamide selective layer. This occurred because more PIP monomers adsorbed onto the porous nanosheets compared to the PES substrate, and the smaller pore size of the nanosheets restricted PIP permeation, promoting the formation of a densely cross-linked polyamide network. By precisely controlling the interlayer and the structure and properties of selective layers, the membrane reached a permeance of 32.7 L m^−2^ h^−1^ bar^−1^ and selectivity of 271.7 for Cl^−^ and SO_4_^2−^. Xu et al. used two-dimensional Al-based nanosheets as an interlayer, applying them onto a nylon membrane surface via vacuum suction. The Al-MOF nanosheets’ high porosity enabled the membrane to achieve a permeance of 42 L m^−2^ h^−1^ bar^−1^ and a Na_2_SO_4_ rejection rate of 97% (Figure 9b) [70]. MOF nanosheets could not only regulate the interfacial polymerization process from beneath the polyamide layer but also act as a switch between the aqueous and organic phases to initiate polymerization. Wang et al. dispersed Cu-BDC nanosheets in hexane, transferred this dispersion into an aqueous phase containing amine monomers, and used solvent evaporation to aid the self-assembly of the nanosheets at the interface [71]. Adding a TMC-containing hexane solution initiated the interfacial polymerization (IP) reaction, where Cu-BDC nanosheets aligned horizontally at the water/hexane interface to speed up amine monomer transport and nanobubble formation. This process resulted in the formation of a wrinkled PA nanofilm. The membrane subsequently achieved high rejection (>90%) for neutral small molecules like boron and N-nitrosodimethylamine (NDMA).

When MOFs serve as an interlayer, the water-phase and organic-phase monomers of interfacial polymerization meet on the MOF surface, embedding MOFs within the selective layer. This configuration makes it challenging to effectively utilize the pore structure of MOFs. In response, Zhao et al. proposed a capillary force-assisted interfacial polymerization method for preparing MOF-PA nanofiltration membranes [72]. This approach involved depositing MOF particles on a PES membrane and then moving it to a polyvinyl alcohol sponge soaked with PIP. Capillary forces drove the PIP solution in the sponge up through MOF particle gaps, reacting with TMC to form a polyamide selective layer. Capillary forces created a PIP concentration gradient at the membrane interface, reducing the PIP levels at the top, which decreased cross-linking and enhanced water flux (Figure 9c). Additionally, due to the high hydrophilicity of the membrane and uniform pore size, it achieved the efficient separation of nutrients and emerging pollutants like perfluoroalkyl substances from wastewater, with a separation factor of around 8.

#### 3.3.2. Doping MOFs into the Aqueous/Organic Phase

Besides serving as an interlayer for selective layer formation control during interfacial polymerization, MOFs could also be dispersed directly into aqueous- or organic-phase solutions, integrating themselves into the selective layer. Jin et al. introduced hydrophilic UiO-66-NH_2_ particles, 15 nm in size, into the aqueous PIP phase, creating an ultrathin selective layer around 20 nm thick [73]. The size compatibility between nanoparticles and the selective layer enabled uniform nanoparticle incorporation, resulting in an ultrathin, defect-free polyamide layer with high water flux. The membrane demonstrated a high flux of 46 L m^−2^ h^−1^ bar^−1^ and approximately 98% retention for Na_2_SO_4_. Apart from preparing small-sized MOF particles for enhanced compatibility with the selective layer, modifying MOF particles is also a prevalent strategy. Fang et al. modified UiO-66-NH_2_ with chitosan and dispersed it into PIP for interfacial polymerization, demonstrating strong anti-fouling properties [74]. Shao et al. selected dopamine and glucose, which are rich in amine and phenolic groups, as aqueous-phase monomers. They introduced UiO-66-NH_2_ to create a ternary biomimetic nanofiltration membrane. Incorporating UiO-66-NH_2_ enhanced the water permeance to 32 L m^−2^ h^−1^ bar^−1^ and preserved high Na_2_SO_4_ retention. This is due to hydrogen bonds between glucose and the amine groups of UiO-66-NH_2_, promoting an even UiO-66-NH_2_ spread and leveraging the MOF porosity for extra molecular transport paths [75]. Moreover, the Zr metal centers in MOFs could also interact with fatty amines. This interaction slows the diffusion rate of amine monomers, leading to selective layers with distinctive morphologies [76]. Jiang et al. introduced UiO-66-NH_2_ particles, around 15 nm in size, into a water solution of TETA. The amine groups of TETA interact with Zr centers of UiO-66-NH_2_, affecting the behavior of the monomer. This interaction decreases the diffusion rate of TETA and increases the amine monomer concentration around the MOF [77,78]. An excessive amine monomer concentration may disrupt the MOF structure by forming intermediate complexes [79]. Han et al. modified MIL-101-NH_2_ particles with TMC and (3-glycidyloxypropyl) triethoxysilane (GPTES) and then dispersed the modified MOF particles in a hexane solution containing TMC [80]. The TFN membrane demonstrated up to 99.0% NaCl retention and approximately 90% removal of small molecules. Both TMC and GPTES modifications to MOFs target the same amine groups. TMC engages these groups in an in situ chemical cross-linking reaction to form amide bonds, thereby enhancing the compatibility and resolving interfacial issues between MOFs and polyamide. GPTES engages in a ring-opening reaction with the epoxy groups and the amines in MOFs. This reaction stabilizes MOF particles in the organic phase as a particle suspension, which inhibits particle aggregation during the interfacial polymerization process. Xu et al. applied post-synthetic metal ion exchange to UiO-66-NH_2_, substituting Zr^4+^ with Ti^3+^ during doping into TMC. This partial replacement increased the negative charge inside MOF pores and enabled faster cation transport. Additionally, the NH_2_ groups in UiO-66-NH_2_ locally reacted with TMC, resulting in chemical crosslinking and giving the membrane a Li^+^ over Mg^2+^ selectivity of 12 [81].

#### 3.3.3. In Situ Cogrowth of MOFs

It is also feasible to synchronize the growth and crystallization of MOFs with the interfacial polymerization process, facilitating the in situ formation of MOFs within the polyamide matrix. This strategy enables a uniform and oriented distribution of MOFs throughout the polyamide network. Fu et al. uniformly sprayed atomized copper ion droplets onto the surface of a mixed solution containing the organic ligand (1,4-benzene dicarboxylic acid, H_2_BDC) and m-phenylenediamine [82]. The resulting MOF was uniformly dispersed within the polymer matrix without defects over a large area, and the membrane exhibited ideal selectivity for Li^+^/Mg^2+^ of 1221.95, with a Li^+^ permeability of 12.54 mol m^−2^ h^−1^. The bifunctional metal ions self-assemble with the organic ligands and stimulate monomer polymerization at the gas–liquid interface, rapidly growing a large-scale interfacial polymerization layer.

Amine monomers could not only in situ form a polyamide network with MOF but also confine the crystallization process of MOFs, resulting in defect-free MOF membranes. By integrating amine monomers with polymers to form nanoreactors, the release of metal ions from the precursors could be regulated, thereby enabling the controlled growth of MOFs. The covalent linkage between the nanoreactors and MOFs maintains both the porous structure of the MOFs and the intercrystalline channels, offering the potential for the fabrication of membranes with high flux and precise sieving capabilities. An et al. confined Zn^2+^ metal ions of ZIF precursors within a mixed solution of PIP and dopamine (DA). Adding Zn^2+^ ions and PIP slowed the reaction and deposition rates of DA on the membrane, creating a 9 nm thick nanoparticle layer [83]. The membrane achieved a permeance of 130 L m^−2^ h^−1^ bar^−1^, successfully retaining over 97% of molecules larger than 1 nm while allowing less than 15% of those smaller than 0.9 nm to pass through (Figure 9d). The team adopted the same strategy in 2021, using n-(2-aminoethyl) piperazine propyl sulfonate and dopamine-derived amphiphilic polymer nanoparticles as templates. However, the complexity of synthesizing amphiphilic polymer nanoparticles may limit its large-scale production [84]. MOF precursors typically contain metal ions and organic ligands, and interfacial polymerization usually involves aqueous-phase amine monomers and an acyl chloride organic phase. Han et al. used in situ synthesis to integrate MOF into polyamide by dissolving the ZIF-8 precursor and PSS in PIP solution and imidazole in a solution of chloroform with acyl chloride, followed by interfacial polymerization to form a selective layer [85]. The in situ synthesis approach ensured the continuous and perfect distribution of ZIF within the selective layer. The membrane flux increased from 10.0 L m^−2^ h^−1^ bar^−1^ to 20.6 L m^−2^ h^−1^ bar^−1^ while maintaining high retention for Na_2_SO_4_.

## 4. Nanofiltration Applications

This section analyzes and compares the performance of the three different types of MOF–polymer membranes in nanofiltration applications (water treatment and organic solvent nanofiltration). It introduces the separation performance and existing problems of these membranes in nanofiltration systems. Through this comparison, we further clarify the structure–performance relationship of polymer–MOF membranes in nanofiltration systems. Due to the limitations of the “trade-off” effect in conventional polymer materials, it is challenging to simultaneously enhance both rejection and flux [86,87]. Incorporating MOFs with high porosity and regular channels can achieve a simultaneous improvement in permeance and rejection, as the MOF channels provide additional transport pathways and selectivity. The synergistic and defect-free integration of polymer and MOF materials is fundamental to achieving high flux and high selectivity.

### 4.1. Water Treatment

The rising demand for freshwater has elevated water scarcity to a global challenge. Water treatment processes include seawater desalination; recovery; and the purification of wastewater containing dyes, heavy metal ions, and organics. The sub-nanometer pore structure of MOF membranes enables high water flux and desalination performance, making them ideal for nanofiltration processes. MOFs can effectively balance high permeance with high rejection. Their stable crystalline structure and ultra-high porosity provide channels that allow water molecules to pass through while retaining small molecules. This characteristic enables MOFs to achieve both high selectivity and high permeability simultaneously. Liu et al. developed MOF-303 directly on an alumina substrate [88]. Based on the hard and soft acids and bases (HSAB) theory, MOF-303 exhibits excellent water stability in water. Possessing a pore size of approximately 0.6 nm, which falls between that of water molecules (0.28 nm) and hydrated ions, the membrane reached retention rates of 93.5% for MgCl_2_ and 96.0% for Na_2_SO_4_. This performance is linked to the dissociated carboxyl groups in the MOF-303 ligands that give the membrane a negatively charged surface, enhancing Na_2_SO_4_ retention. Liu et al. synthesized UiO-66 on an alumina substrate, achieving retention rates of 86.3% for Ca^2+^, 98% for Mg^2+^, and 99.3% for Al^3+^ over 170 h [35]. The long-term stability could be attributed to the high stability of UIO-66. Jian et al. produced two-dimensional Al-MOF nanosheets on an AAO substrate, reaching nearly 100% removal of inorganic salts such as NaCl and KCl [89]. The exceptional retention and stability are due to the strong self-locking structure formed by p–p interactions among MOF nanosheets, with the performance stability exceeding 750 h. The crystal structure of MOFs crucially impacts their separation performance, and defect engineering allows pore size adjustment to enhance the separation capabilities. Wang et al. utilized post-synthetic ligand repair on defect-rich UiO-66, altering its pore size from 1.26 nm to 0.4 nm and boosting NaCl retention from 26% to 45% [90]. MMMs with MOFs typically show suboptimal ion separation performance. Zhang et al. evaluated several MOFs embedded in PVC and discovered that UiO-66-SO_3_H@PVC exhibited the highest Li^+^/Mg^2+^ selectivity, attributed to its sulfonate groups. However, its selectivity, at a value of 4, was considerably lower than that of MOF polycrystalline membranes. This may be due to the poor binding force between the MOF and the polymer in MMMs, leading to more interfacial voids [91]. The following table summarizes the performance of the three MOF membrane types in water treatment systems, as introduced earlier (Table 1). In desalination applications, MOF polycrystalline membranes achieve retention rates exceeding 95%. However, their compact structure results in increased membrane thickness and reduced flux, ranging from 0.1 to 4 L m^−2^ h^−1^ bar^−1^. For ion separation, MOF polycrystalline membranes demonstrate excellent ion separation capabilities due to uniform pores and the ability to specifically bind ions. MMMs applied to water treatment systems struggle with effective ion rejection and separation due to interfacial defects. TFN membranes, prepared through MOF incorporation and interfacial polymerization, exhibit the most outstanding performance among the three membranes. The polyamide layer, inherently containing extensive sub-nanometer channels, is generated via interfacial polymerization. Additionally, MOFs enhance this structure by regulating the polymerization process and contributing their sub-nanometer channels to facilitate material transport.

### 4.2. Organic Solvent Nanofiltration

**Figure 10:**
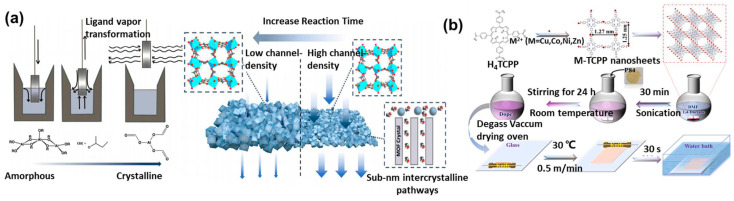
Applications of MOF-based membranes in organic solvent nanofiltration. (**a**) In situ preparation of Al-MOF membranes for organic solvent systems [93], and (**b**) fabrication of MOF mixed-matrix membranes for organic solvent nanofiltration systems [94].

Organic solvent separation is vital in industries like biorefining, petrochemicals, and pharmaceuticals, forming a crucial aspect of industrial manufacturing. The primary challenge in organic solvent nanofiltration (OSN) lies in developing membranes that are chemically stable in organic solvents. MOFs have attracted widespread attention as promising materials for OSN membranes due to their stability in common organic solvents. Zhao et al. processed an amorphous Al precursor with formic acid via a sol–gel method to produce an Al-MOF [93]. This membrane demonstrates precise molecular sieving capabilities in organic solvent systems. By adjusting the reaction time, the pore size of the membrane could be fine-tuned from 0.6 to 2 nm, enabling the molecular weight cutoff to be set between 300 and 650 Da and achieving ethanol permeability of 0.8–22 L m^−2^ h^−1^ bar^−1^ (Figure 10a). However, MMMs also encounter poor retention in OSN systems due to interfacial defects. Yao et al. incorporated Cu-TCPP as the filler into a P84 membrane substrate to create a MOF mixed-matrix membrane [94]. They observed that the dispersion state of the filler greatly affects the performance of MMM, with fully dispersed Cu-TCPP almost doubling the permeance compared to partial dispersion, though retention for bright blue R (Mw = 825.97) stayed nearly constant at 95.7% (Figure 10b). Ma et al. synthesized UiO-66 on a solvent-resistant substrate via a multi-growth method. As this method did not yield a complete and defect-free crystal structure, the membrane retained 96.33% of the large molecular dye Bengal rose (Mw = 1017.64), with an ethanol permeation flux of 0.88 L m^−2^ h^−1^ bar^−1^. Table 2 summarizes the performance of three MOF membrane types in organic solvent systems, as introduced before. Similar to their performance in water treatment, MMM membranes in organic solvent systems exhibit low flux and limited separation precision. In contrast, MOF polycrystalline membranes and TFN membranes stand out in separation efficiency due to the distinctive narrow pores of MOFs.

## 5. Summary and Outlook

This review introduces the synergy between polymers and MOFs in membrane construction, focusing on polymer-enhanced MOF functionality and high-performance membrane construction with sub-nanometer channels. It discusses the roles of polymers in MOF surface modification, growth regulation, and support carriers. In the future, it will be essential to address several key considerations to improve the separation performance and functionalize MOF–polymer composite membranes.

(1) Interaction mechanisms between polymers and MOFs

Polymers could regulate the growth of MOFs during synthesis by controlling the number and angles of connections between metal clusters and organic ligands, thereby influencing the growth process of MOFs. Although researchers have employed density functional theory simulations to elucidate the role of polymers in MOF synthesis, direct observation or detection of the roles of polymers during MOF synthesis remains exceedingly challenging. Future research should utilize sub-nanometer-scale characterization or detection techniques (such as in situ TEM and two-dimensional NMR) to establish interaction models between polymers and MOFs and profoundly analyze their growth regulation mechanisms.

(2) Coordinated regulation of MOF crystallization and polymer membrane formation

The crystallization of MOFs often demands harsh conditions such as high temperatures, high pressures, and organic solvents, which typically exceed the tolerance of polymer substrates and thereby hinder the collaborative construction of high-performance functional materials. To address this, mitigating the harsh conditions of MOF growth to better align with the processes for forming polymer membranes could be a promising direction. Studies have shown that polymers, due to their strong compatibility, could precisely embed various ligands or metal ions into MOF structural units and even replace organic solvents as growth media for MOFs. Furthermore, the growth of MOFs generally requires acids or bases as regulators, which adversely affect the construction of polymer membranes. Consequently, exploring the use of functional polymers to replace these acids or bases during MOF synthesis represents a worthwhile direction for research. Additionally, the in situ growth strategy of polymers and MOFs, capable of significantly reducing the MOF synthesis time and addressing issues of dispersibility and interfacial compatibility, should be further developed to enhance the processability of MOFs.

(3) Scaling up the fabrication of the MOF-based membrane for practical applications

A major barrier to the large-scale application of MOF–polymer materials is the high cost of MOF production. By deploying these materials in high-value fields such as biomedicine and chiral separation, the associated costs could be offset. Meanwhile, the inherent brittleness of MOF crystals often leads to defects at crystal interfaces during large-scale production. Emerging research is focused on transforming MOFs into an amorphous and glassy state to eliminate crystal face defects and achieve self-supporting MOFs. These research directions are worth close attention.

## Figures and Tables

**Table 1:** Comparison of different MOF-based membranes for water treatment applications.

Membrane	Type	Permeance (L m^−2^ h^−1^ bar^−1^)	Rejection or Selectivity	Ref
MOF-808	PMOF	4.37	287.3 (Salt/dye)	[36]
UiO-67	PMOF	/	159.4 (Li^+^/Mg^2+^)	[37]
HKUST-1	PMOF	/	10,296 (Li^+^/Mg^2+^)	[39]
NUS-8	PMOF	3	98%, MgCl_2_	[49]
MOF-303	PMOF	3.0	96.0%, Na_2_SO_4_	[88]
UiO-66	PMOF	0.14	98%, MgCl_2_	[92]
UiO-66	PMOF	0.285	45%, NaCl	[90]
UiO-66-SO_3_H	MMM	/	4 (Li^+^/Mg^2+^)	[91]
MIL-101-NH_2_	TFN	20.0	99.0% (Methyl blue, MW = 799.80)	[67]
Cu-TCPP	TFN	32.7	271.7 (Cl^−^/SO_4_^2−^)	[69]
Al-MOF	TFN	42.4	97.0%, Na_2_SO_4_	[70]
Cu-BDC	TFN	4.0	>90%, (Boron and NDMA)	[71]
UiO-66-NH_2_	TFN	46.0	97.1%, Na_2_SO_4_	[73]
UiO-66-NH_2_	TFN	31.5	99.9%, Na_2_SO_4_	[75]
Cu-BDC	TFN	/	1221.95 (Li^+^/Mg^2+^)	[82]
ZIF-8	TFN	130.0	97% (>1 nm molecule)	[83]
ZIF-8	TFN	20.6	96.5%, Na_2_SO_4_	[85]

Note: MOF polycrystalline membranes are abbreviated as PMOF in the table.

**Table 2:** Performance comparison of MOF-based membranes in organic solvent nanofiltration.

Membrane	Type	Permeance (L m^−2^ h^−1^ bar^−1^)	Rejection or Selectivity	Ref
Al-MOF	PMOF	EtOH, 0.8-22	99% (300–650 Da molecule)	[93]
Cu-TCPP	MMM	EtOH, 2.82	95.7% (Brilliant Blue R, Mw = 825.97)	[94]
UiO-66−NH_2_	MMM	EtOH, 0.88	96.33% (Rose Bengal, Mw = 1017.64)	[95]
Fe-TCPP	TFN	MeOH, 120	97.0% (Acid Fuchsin, Mw = 585.54)	[68]
UiO-66−NH_2_	TFN	EtOH, 30.2	98%, (Rose Bengal, Mw = 1017.64)	[75]

## Data Availability

The data are available from the corresponding author on reasonable request.

## References

[1] Jiang Z., Dong R., Evans A.M., Biere N., Ebrahim M.A., Li S., Anselmetti D., Dichtel W.R., Livingston A.G. (2022). Aligned macrocycle pores in ultrathin films for accurate molecular sieving. Nature.

[2] Liu M.-L., Zhang C.-X., Tang M.-J., Sun S.-P., Xing W., Lee Y.M. (2023). Evolution of functional nanochannel membranes. Prog. Mater. Sci..

[3] Wang Z., Luo X., Zhang J., Zhang F., Fang W., Jin J. (2023). Polymer membranes for organic solvent nanofiltration: Recent progress, challenges and perspectives. Adv. Membr..

[4] Cheng Y., Datta S.J., Zhou S., Jia J., Shekhah O., Eddaoudi M. (2022). Advances in metal–organic framework-based membranes. Chem. Soc. Rev..

[5] Dou H., Xu M., Wang B., Zhang Z., Wen G., Zheng Y., Luo D., Zhao L., Yu A., Zhang L. (2021). Microporous framework membranes for precise molecule/ion separations. Chem. Soc. Rev..

[6] Wang X., Ma Q., Cheng J., He D., Zhang L., Lu P., Jin H., Choi J., Li Y. (2022). Crystallization-controlled defect minimization of a ZIF-67 membrane for the robust separation of propylene and propane. J. Membr. Sci..

[7] Wang X., Lyu Q., Tong T., Sun K., Lin L.-C., Tang C.Y., Yang F., Guiver M.D., Quan X., Dong Y. (2022). Robust ultrathin nanoporous MOF membrane with intra-crystalline defects for fast water transport. Nat. Commun..

[8] Feng Y., Wang H., Yao J. (2021). Synthesis of 2D nanoporous zeolitic imidazolate framework nanosheets for diverse applications. Coord. Chem. Rev..

[9] Uemura T., Uchida N., Asano A., Saeki A., Seki S., Tsujimoto M., Isoda S., Kitagawa S. (2012). Highly Photoconducting π-Stacked Polymer Accommodated in Coordination Nanochannels. J. Am. Chem. Soc..

[10] Yang S.L., Karve V.V., Justin A., Kochetygov I., Espín J., Asgari M., Trukhina O., Sun D.T., Peng L., Queen W.L. (2021). Enhancing MOF performance through the introduction of polymer guests. Coord. Chem. Rev..

[11] Peng L., Yang S.L., Sun D.T., Asgari M., Queen W.L. (2018). MOF/polymer composite synthesized using a double solvent method offers enhanced water and CO adsorption properties. Chem. Commun..

[12] Huang G., Yang Q., Xu Q., Yu S.H., Jiang H.L. (2016). Polydimethylsiloxane Coating for a Palladium/MOF Composite: Highly Improved Catalytic Performance by Surface Hydrophobization. Angew. Chem. Int. Ed..

[13] Wen L., Sun K., Liu X., Yang W., Li L., Jiang H.L. (2023). Electronic State and Microenvironment Modulation of Metal Nanoparticles Stabilized by MOFs for Boosting Electrocatalytic Nitrogen Reduction. Adv. Mater..

[14] Koutsianos A., Pallach R., Frentzel-Beyme L., Das C., Paulus M., Sternemann C., Henke S. (2023). Breathing porous liquids based on responsive metal-organic framework particles. Nat. Commun..

[15] He S., Chen L., Cui J., Yuan B., Wang H., Wang F., Yu Y., Lee Y., Li T. (2019). General Way To Construct Micro- and Mesoporous Metal–Organic Framework-Based Porous Liquids. J. Am. Chem. Soc..

[16] Xu M., Li D., Sun K., Jiao L., Xie C., Ding C., Jiang H.L. (2021). Interfacial Microenvironment Modulation Boosting Electron Transfer between Metal Nanoparticles and MOFs for Enhanced Photocatalysis. Angew. Chem. Int. Ed..

[17] Zuo Q., Liu T., Chen C., Ji Y., Gong X., Mai Y., Zhou Y. (2019). Ultrathin Metal–Organic Framework Nanosheets with Ultrahigh Loading of Single Pt Atoms for Efficient Visible-Light-Driven Photocatalytic H2 Evolution. Angew. Chem. Int. Ed..

[18] Zimpel A., Al Danaf N., Steinborn B., Kuhn J., Hohn M., Bauer T., Hirschle P., Schrimpf W., Engelke H., Wagner E. (2019). Coordinative Binding of Polymers to Metal-Organic Framework Nanoparticles for Control of Interactions at the Biointerface. ACS Nano.

[19] Lyu F.J., Zhang Y.F., Zare R.N., Ge J., Liu Z. (2014). One-Pot Synthesis of Protein-Embedded Metal-Organic Frameworks with Enhanced Biological Activities. Nano Lett..

[20] Albayati N., Kadhom M. (2020). Preparation of functionalised UiO-66 metal–organic frameworks (MOFs) nanoparticles using deep eutectic solvents as a benign medium. Micro Nano Lett..

[21] Wang C.Z., Tadepalli S., Luan J.Y., Liu K.K., Morrissey J.J., Kharasch E.D., Naik R.R., Singamaneni S. (2017). Metal-Organic Framework as a Protective Coating for Biodiagnostic Chips. Adv. Mater..

[22] Liang K., Ricco R., Doherty C.M., Styles M.J., Bell S., Kirby N., Mudie S., Haylock D., Hill A.J., Doonan C.J. (2015). Biomimetic mineralization of metal-organic frameworks as protective coatings for biomacromolecules. Nat. Commun..

[23] Li P., Cheng F.F., Xiong W.W., Zhang Q.C. (2018). New synthetic strategies to prepare metal-organic frameworks. Inorg. Chem. Front..

[24] Gao J.K., He M., Lee Z.Y., Cao W.F., Xiong W.W., Li Y.X., Ganguly R., Wu T., Zhang Q.C. (2013). A surfactant-thermal method to prepare four new three-dimensional heterometal-organic frameworks. Dalton Trans..

[25] Westendorff K.S., Paolucci C., Giri G. (2021). Polymer-induced polymorphism in a Zn-based metal organic framework. Chem. Commun..

[26] Yao J., He M., Wang K., Chen R., Zhong Z., Wang H. (2013). High-yield synthesis of zeolitic imidazolate frameworks from stoichiometric metal and ligand precursor aqueous solutions at room temperature. CrystEngComm.

[27] Peng L., Zhang X., Sun Y., Xing Y., Li C. (2020). Heavy metal elimination based on metal organic framework highly loaded on flexible nanofibers. Environ. Res..

[28] Xu S., Liang J., Mohammad M.I.B., Lv D., Cao Y., Qi J., Liang K., Ma J. (2021). Biocatalytic metal–organic framework membrane towards efficient aquatic micropollutants removal. Chem. Eng. J..

[29] Uemura T., Hoshino Y., Kitagawa S., Yoshida K., Isoda S. (2006). Effect of organic polymer additive on crystallization of porous coordination polymer. Chem. Mater..

[30] Jiang D., Mallat T., Krumeich F., Baiker A. (2011). Polymer-assisted synthesis of nanocrystalline copper-based metal–organic framework for amine oxidation. Catal. Commun..

[31] Wang C., Zhang H., Wang Y., Wu J., Kirlikovali K.O., Li P., Zhou Y., Farha O.K. (2022). A General Strategy for the Synthesis of Hierarchically Ordered Metal–Organic Frameworks with Tunable Macro-, Meso-, and Micro-Pores. Small.

[32] Xu Z., Liu C., Xiao L., Meng Q., Zhang G. (2024). Metal-organic frameworks-based mixed matrix pervaporation membranes for recovery of organics. Adv. Membr..

[33] Liu Y., Chen H., Li T., Ren Y., Wang H., Song Z., Li J., Zhao Q., Li J., Li L. (2023). Balancing the Crystallinity and Film Formation of Metal-Organic Framework Membranes through In Situ Modulation for Efficient Gas Separation. Angew. Chem. Int. Ed. Engl..

[34] Li Y.S., Liang F.Y., Bux H., Feldhoff A., Yang W.S., Caro J. (2010). Molecular sieve membrane: Supported metal-organic framework with high hydrogen selectivity. Angew. Chem. Int. Ed. Engl..

[35] Huang A.S., Dou W., Caro J. (2010). Steam-Stable Zeolitic Imidazolate Framework ZIF-90 Membrane with Hydrogen Selectivity through Covalent Functionalization. J. Am. Chem. Soc..

[36] Wu M., Sun Y., Ji T., Yu K., Liu L., He Y., Yan J., Meng S., Hu W., Fan X. (2023). Fabrication of water-stable MOF-808 membrane for efficient salt/dye separation. J. Membr. Sci..

[37] Xu R., Kang Y., Zhang W., Zhang X., Pan B. (2021). Oriented UiO-67 Metal–Organic Framework Membrane with Fast and Selective Lithium-Ion Transport. Angew. Chem. Int. Ed..

[38] Wang J.Y., Wang Y., Liu Y.T., Wu H., Zhao M.G., Ren Y.X., Pu Y.C.A., Li W.P., Wang S.Y., Song S.Q. (2022). Ultrathin ZIF-8 Membrane through Inhibited Ostwald Ripening for High-Flux CH/CH Separation. Adv. Funct. Mater..

[39] Guo Y., Ying Y., Mao Y., Peng X., Chen B. (2016). Polystyrene Sulfonate Threaded through a Metal–Organic Framework Membrane for Fast and Selective Lithium-Ion Separation. Angew. Chem. Int. Ed..

[40] Zhou Y., Zhang X.-F., Yao J., Wang H. (2022). Contra-diffusion synthesis of metal-organic framework separation membranes: A review. Sep. Purif. Technol..

[41] Yao J.F., Dong D.H., Li D., He L., Xu G.S., Wang H.T. (2011). Contra-diffusion synthesis of ZIF-8 films on a polymer substrate. Chem. Commun..

[42] You X., Wu H., Zhang R., Su Y., Cao L., Yu Q., Yuan J., Xiao K., He M., Jiang Z. (2019). Metal-coordinated sub-10 nm membranes for water purification. Nat. Commun..

[43] Li B., You X., Wu H., Li R., Xiao K., Ren Y., Wang H., Song S., Wang Y., Pu Y. (2022). A facile metal ion pre-anchored strategy for fabrication of defect-free MOF membranes on polymeric substrates. J. Membr. Sci..

[44] Yu C., Cen X., Zhang Z., Sun Y., Xue W., Qiao Z., Guiver M.D., Zhong C. (2023). Step-Nucleation In Situ Self-Repair to Prepare Rollable Large-Area Ultrathin MOF Membranes. Adv. Mater..

[45] Yu C., Jia Y., Fang K., Qin Y., Deng N., Liang Y. (2022). Preparation hierarchical porous MOF membranes with island-like structure for efficient gas separation. J. Membr. Sci..

[46] Chen J., Wu X., Chen C., Chen Y., Li W., Wang J. (2022). Secondary-assembled defect-free MOF membrane via triple-needle electrostatic atomization for highly stable and selective organics permeation. J. Membr. Sci..

[47] Xu L.H., Li S.H., Mao H., Li Y., Zhang A.S., Wang S., Liu W.M., Lv J., Wang T., Cai W.W. (2022). Highly flexible and superhydrophobic MOF nanosheet membrane for ultrafast alcohol-water separation. Science.

[48] Hu Z., Mahdi E.M., Peng Y., Qian Y., Zhang B., Yan N., Yuan D., Tan J.-C., Zhao D. (2017). Kinetically controlled synthesis of two-dimensional Zr/Hf metal–organic framework nanosheets via a modulated hydrothermal approach. J. Mater. Chem. A.

[49] Yuan H., Liu G., Qiao Z., Li N., Buenconsejo P.J.S., Xi S., Karmakar A., Li M., Cai H., Pennycook S.J. (2021). Solution-Processable Metal–Organic Framework Nanosheets with Variable Functionalities. Adv. Mater..

[50] Yuan H.Y., Li K.R., Shi D.C., Yang H., Yu X., Fan W.D., Buenconsejo P.J.S., Zhao D. (2023). Large-Area Fabrication of Ultrathin Metal-Organic Framework Membranes. Adv. Mater..

[51] Wang Z., Wang W., Zeng T., Ma D., Zhang P., Zhao S., Yang L., Zou X., Zhu G. (2021). Covalent-Linking-Enabled Superior Compatibility of ZIF-8 Hybrid Membrane for Efficient Propylene Separation. Adv. Mater..

[52] Cseri L., Hardian R., Anan S., Vovusha H., Schwingenschlögl U., Budd P.M., Sada K., Kokado K., Szekely G. (2021). Bridging the interfacial gap in mixed-matrix membranes by nature-inspired design: Precise molecular sieving with polymer-grafted metal–organic frameworks. J. Mater. Chem. A.

[53] Wang X., Wu L., Li N., Fan Y. (2021). Sealing Tröger base/ZIF-8 mixed matrix membranes defects for improved gas separation performance. J. Membr. Sci..

[54] Wu C., Zhang K., Wang H., Fan Y., Zhang S., He S., Wang F., Tao Y., Zhao X., Zhang Y.-B. (2020). Enhancing the Gas Separation Selectivity of Mixed-Matrix Membranes Using a Dual-Interfacial Engineering Approach. J. Am. Chem. Soc..

[55] Su Y., Li D., Shan M., Feng X., Gascon J., Wang Y., Zhang Y. (2024). Uniformly Distributed Mixed Matrix Membranes via a Solution Processable Strategy for Propylene/Propane Separation. Angew. Chem. Int. Ed..

[56] Chen K., Ni L., Zhang H., Li L., Guo X., Qi J., Zhou Y., Zhu Z., Sun X., Li J. (2023). Phenolic resin regulated interface of ZIF-8 based mixed matrix membrane for enhanced gas separation. J. Membr. Sci..

[57] Carja I.-D., Tavares S.R., Shekhah O., Ozcan A., Semino R., Kale V.S., Eddaoudi M., Maurin G. (2021). Insights into the Enhancement of MOF/Polymer Adhesion in Mixed-Matrix Membranes via Polymer Functionalization. ACS Appl. Mater. Interfaces.

[58] Lee T.H., Lee B.K., Yoo S.Y., Lee H., Wu W.-N., Smith Z.P., Park H.B. (2023). PolyMOF nanoparticles constructed from intrinsically microporous polymer ligand towards scalable composite membranes for CO_2_ separation. Nat. Commun..

[59] Seoane B., Sebastián V., Téllez C., Coronas J. (2013). Crystallization in THF: The possibility of one-pot synthesis of mixed matrix membranes containing MOF MIL-68(Al). CrystEngComm.

[60] Li S., Sun Y.J., Wang Z.X., Jin C.G., Yin M.J., An Q.F. (2023). Rapid Fabrication of High-Permeability Mixed Matrix Membranes at Mild Condition for CO2 Capture. Small.

[61] Chen G., Chen C., Guo Y., Chu Z., Pan Y., Liu G., Liu G., Han Y., Jin W., Xu N. (2023). Solid-solvent processing of ultrathin, highly loaded mixed-matrix membrane for gas separation. Science.

[62] He S., Zhu B., Jiang X., Han G., Li S., Lau C.H., Wu Y., Zhang Y., Shao L. (2022). Symbiosis-inspired de novo synthesis of ultrahigh MOF growth mixed matrix membranes for sustainable carbon capture. Proc. Natl. Acad. Sci. USA.

[63] Morgan P.W., Kwolek S.L. (2003). Interfacial polycondensation. II. Fundamentals of polymer formation at liquid interfaces. J. Polym. Sci..

[64] Kadhom M., Deng B. (2019). Synthesis of high-performance thin film composite (TFC) membranes by controlling the preparation conditions: Technical notes. J. Water Process Eng..

[65] Shu L., Peng Y., Song H., Zhu C., Yang W. (2023). Modular Customization and Regulation of Metal-Organic Frameworks for Efficient Membrane Separations. Angew. Chem. Int. Ed. Engl..

[66] Karan S., Jiang Z.W., Livingston A.G. (2015). Sub-10 nm polyamide nanofilms with ultrafast solvent transport for molecular separation. Science.

[67] Zhao Z., Shehzad M.A., Wu B., Wang X., Yasmin A., Zhu Y., Wang X., He Y., Ge L., Li X. (2021). Spray-deposited thin-film composite MOFs membranes for dyes removal. J. Membr. Sci..

[68] Hussain S., Bahadar S., Wang G., Zhu L., Ye Z., Peng X. (2022). Photothermal-driven interfacial-polymerized ultrathin polyamide selective layer for nanofiltration. Chem. Eng. J..

[69] Cheng P., Liu Y., Wang X., Fan K., Li P., Xia S. (2022). Regulating interfacial polymerization via constructed 2D metal-organic framework interlayers for fabricating nanofiltration membranes with enhanced performance. Desalination.

[70] Jin X.-G., Liang X.-K., Liu J.-H., Mo J.-W., Ren T.-X., Ma X.-H., Xu Z.-L. (2023). Development of high permeability nanofiltration membranes through porous 2D MOF nanosheets. Chem. Eng. J..

[71] Wen Y., Dai R.B., Li X.S., Zhang X.R., Cao X.Z., Wu Z.C., Lin S.H., Tang C.Y., Wang Z.W. (2022). Metal-organic framework enables ultraselective polyamide membrane for desalination and water reuse. Sci. Adv..

[72] Zhao Y., Tong X., Kim J., Tong T., Huang C.-H., Chen Y. (2022). Capillary-Assisted Fabrication of Thin-Film Nanocomposite Membranes for Improved Solute–Solute Separation. Environ. Sci. Technol..

[73] Gong Y., Gao S., Tian Y., Zhu Y., Fang W., Wang Z., Jin J. (2020). Thin-film nanocomposite nanofiltration membrane with an ultrathin polyamide/UIO-66-NH2 active layer for high-performance desalination. J. Membr. Sci..

[74] Fang S.-Y., Gong J.-L., Tang L., Li J., Qin M., Zhou H.-Y., Tang L.-X., Zhao J. (2023). Thin-Film Nanocomposite Membranes with Nature-Inspired MOFs Incorporated for Removing Fluoroquinolone Antibiotics. ACS Appl. Mater. Interfaces.

[75] Zhang Y., Cheng X., Jiang X., Urban J.J., Lau C.H., Liu S., Shao L. (2020). Robust natural nanocomposites realizing unprecedented ultrafast precise molecular separations. Mater. Today.

[76] Jiao C., Song X., Zhang X., Sun L., Jiang H. (2021). MOF-Mediated Interfacial Polymerization to Fabricate Polyamide Membranes with a Homogeneous Nanoscale Striped Turing Structure for CO2/CH4 Separation. ACS Appl. Mater. Interfaces.

[77] Tan Z., Chen S.F., Peng X.S., Zhang L., Gao C.J. (2018). Polyamide membranes with nanoscale Turing structures for water purification. Science.

[78] Tang M.J., Liu M.L., Wang D.A., Shao D.D., Wang H.J., Cui Z.L., Cao X.L., Sun S.P. (2020). Precisely Patterned Nanostrand Surface of Cucurbituril [6]-Based Nanofiltration Membranes for Effective Alcohol-Water Condensation. Nano Lett..

[79] Kuwahara Y., Kang D.Y., Copeland J.R., Brunelli N.A., Didas S.A., Bollini P., Sievers C., Kamegawa T., Yamashita H., Jones C.W. (2012). Dramatic Enhancement of CO Uptake by Poly(ethyleneimine) Using Zirconosilicate Supports. J. Am. Chem. Soc..

[80] Han G., Studer R.M., Lee M., Rodriguez K.M., Teesdale J.J., Smith Z.P. (2023). Post-synthetic modification of MOFs to enhance interfacial compatibility and selectivity of thin-film nanocomposite (TFN) membranes for water purification. J. Membr. Sci..

[81] Xu T., Sheng F., Wu B., Shehzad M.A., Yasmin A., Wang X., He Y., Ge L., Zheng X., Xu T. (2020). Ti-exchanged UiO-66-NH2–containing polyamide membranes with remarkable cation permselectivity. J. Membr. Sci..

[82] Zhang B., Dai X., Wei N., Cui X., Fan F., Zhang J., Zhang D., Meng F., Qi W., Fu Y. (2023). Fabrication of Oriented MOF-Based Mixed Matrix Membrane via Ion-Induced Synchronous Synthesis. Small.

[83] Ji Y.-L., Gu B.-X., Huo H.-Q., Xie S.-J., Peng H., Zhang W.-H., Yin M.-J., Xiong B., Lu H., Villalobos L.F. (2024). Roll-to-roll fabrication of large-area metal–organic framework-based membranes for high-performance aqueous separations. Nat. Water.

[84] Ji Y.L., Gu B.X., Xie S.J., Yin M.J., Qian W.J., Zhao Q., Hung W.S., Lee K.R., Zhou Y., An Q.F. (2021). Superfast Water Transport Zwitterionic Polymeric Nanofluidic Membrane Reinforced by Metal–Organic Frameworks. Adv. Mater..

[85] Jia M.-M., Feng J.-H., Shao W., Chen Z., Yu J.-R., Sun J.-J., Wu Q.-Y., Li Y., Xue M., Chen X.-M. (2024). In-situ interfacial synthesis of metal-organic framework/polyamide thin-film nanocomposite membranes with elevated nanofiltration performances. J. Membr. Sci..

[86] Meng Q.-W., Cheng L., Ge Q. (2023). Recent advances and future challenges of polyamide-based chlorine-resistant membrane. Adv. Membr..

[87] Peng H.Y., Lau S.K., Yong W.F. (2024). Recent advances of thin film composite nanofiltration membranes for Mg2+/Li+ separation. Adv. Membr..

[88] Cong S., Yuan Y., Wang J., Wang Z., Kapteijn F., Liu X. (2021). Highly Water-Permeable Metal–Organic Framework MOF-303 Membranes for Desalination. J. Am. Chem. Soc..

[89] Jian M.P., Qiu R.S., Xia Y., Lu J., Chen Y., Gu Q.F., Liu R.P., Hu C.Z., Qu J.H., Wang H.T. (2020). Ultrathin water-stable metal-organic framework membranes for ion separation. Sci. Adv..

[90] Wang X., Zhai L., Wang Y., Li R., Gu X., Yuan Y.D., Qian Y., Hu Z., Zhao D. (2017). Improving Water-Treatment Performance of Zirconium Metal-Organic Framework Membranes by Postsynthetic Defect Healing. ACS Appl. Mater. Interfaces.

[91] Zhang C., Mu Y., Zhang W., Zhao S., Wang Y. (2020). PVC-based hybrid membranes containing metal-organic frameworks for Li+/Mg2+ separation. J. Membr. Sci..

[92] Liu X.L., Demir N.K., Wu Z.T., Li K. (2015). Highly Water-Stable Zirconium Metal Organic Framework UiO-66 Membranes Supported on Alumina Hollow Fibers for Desalination. J. Am. Chem. Soc..

[93] Shi D., Li H., Yu X., Zhang Z., Yuan Y.D., Fan W., Yuan H., Ying Y., Yang H., Shang C. (2023). Intercrystalline Channels at Subnanometer Scale for Precise Molecular Nanofiltration. J. Am. Chem. Soc..

[94] Yao A., Hua D., Zhao F., Zheng D., Pan J., Hong Y., Liu Y., Rao X., Zhou S., Zhan G. (2022). Integration of P84 and porphyrin–based 2D MOFs (M−TCPP, M = Zn, Cu, Co, Ni) for mixed matrix membranes towards enhanced performance in organic solvent nanofiltration. Sep. Purif. Technol..

[95] Ma D., Han G., Gao Z.F., Chen S.B. (2019). Continuous UiO-66-Type Metal–Organic Framework Thin Film on Polymeric Support for Organic Solvent Nanofiltration. ACS Appl. Mater. Interfaces.

